# The Acute Phase Protein Serum Amyloid A Induces Lipolysis and Inflammation in Human Adipocytes through Distinct Pathways

**DOI:** 10.1371/journal.pone.0034031

**Published:** 2012-04-19

**Authors:** Aurélie Faty, Pascal Ferré, Stéphane Commans

**Affiliations:** 1 Metabolic Pathways Center of Excellence in Drug Discovery, GlaxoSmithKline, Les Ulis, France; 2 INSERM, UMR-S 872 (Eq 8), Cordeliers Research Center, Paris, France; 3 Université Pierre and Marie Curie-Paris6, UMR-S 872, Cordeliers Research Center, Paris, France; Pennington Biomedical Research Center, United States of America

## Abstract

**Background:**

The acute phase response (APR) is characterized by alterations in lipid and glucose metabolism leading to an increased delivery of energy substrates. In adipocytes, there is a coordinated decrease in Free Fatty acids (FFAs) and glucose storage, in addition to an increase in FFAs mobilization. Serum Amyloid A (SAA) is an acute phase protein mainly associated with High Density Lipoproteins (HDL). We hypothesized that enrichment of HDL with SAA, during the APR, could be implicated in the metabolic changes occurring in adipocytes.

**Methodology/Principal Findings:**

*In vitro* differentiated human adipocytes (hMADS) were treated with SAA enriched HDL or recombinant SAA and the metabolic phenotype of the cells analyzed. In hMADS, SAA induces an increased lipolysis through an ERK dependent pathway. At the molecular level, SAA represses PPARγ2, C/EBPα and SREBP-1c gene expression, three transcription factors involved in adipocyte differentiation or lipid synthesis. In addition, the activation of the NF-κB pathway by SAA leads to the induction of pro-inflammatory cytokines and chemokines, as in the case of immune cells. These latter findings were replicated in freshly isolated mature human adipocytes.

**Conclusions/Significance:**

Besides its well-characterized role in cholesterol metabolism, SAA has direct metabolic effects on human adipocytes. These metabolic changes could be at least partly responsible for alterations of adipocyte metabolism observed during the APR as well as during pathophysiological conditions such as obesity and conditions leading to insulin resistant states.

## Introduction

The acute phase response (APR) induced during infection or inflammation, is an early and highly complex reaction of the host, which protects it from further injury. The APR is characterized by an increased resting energy expenditure, extensive protein and fat catabolism, negative nitrogen balance, hyperglycemia and hypertriglyceridemia [1], [2]. The alterations in lipid and glucose metabolism are linked to an impairment of insulin sensitivity [3]. Insulin resistance in muscles, liver and adipose tissue ensures a high flow of glucose and Free Fatty Acids (FFAs) to the predominantly energy-consuming cells, such as the inflammatory and immune cells [4]. Glucose and FFAs uptake by muscles are decreased while hepatic glucose and Very Low Density Lipoproteins (VLDL) productions are increased [1]. The hepatic-linked hyperglycemia and hypertriglyceridemia produced throughout the APR could be a consequence of alterations of adipose tissue metabolism [5]. Indeed, in adipocytes, there is a coordinated decrease in FFA storage and an increase in glycerol and FFA mobilization through stimulation of lipolysis, which could potentially affect hepatic metabolism [1]. The “insulin resistant” metabolic response observed during the APR shares some similarities with the metabolic abnormalities linked to a variety of very common disorders, such as diabetes, chronic renal failure, atherosclerosis, obesity and metabolic syndrome [6]–[9]. Many of these disorders are concomitant with a low grade inflammation with changes in circulating proteins consistent with the profile observed during the APR, although less pronounced.

Serum Amyloid A (SAA) is one of the major acute-phase proteins predominantly produced by the liver [10]. The circulating concentration of SAA protein is increased by 1000-fold within 24 to 48 h following infection/inflammation from a basal level of 5–8 µg/mL. SAA is primarily transported in the plasma by High Density Lipoproteins (HDL), for which it has a high affinity [11]. SAA is thought to be delivered by SAA-enriched HDL (saaHDL) to the sites of infection where it can prime monocytes through its cytokine-like properties [12]–[14]. In immune cells, SAA has been shown to induce an inflammatory response through a variety of receptors [15], [16]. Plasmatic SAA is also more modestly elevated (15–50 µg/mL) in chronic disorders characterized by increased inflammation such as rheumatic diseases, atherosclerosis, diabetes and obesity [17]–[20]. It has been shown that in obese patients, enlarged adipocytes are a source of plasma SAA [21]. In addition, recent data in stromavascular cells from human breast differentiated in vitro in adipocytes, suggest that SAA could promote lipolysis by decreasing perilipin expression and increasing hormone sensitive lipase expression [22]. In porcine adipocytes, SAA induces lipolysis by phosphorylating hormone sensitive lipase (HSL) and by downregulating perilipin through extracellular signal-regulated kinase (ERK) and protein kinase A (PKA) dependent pathways [23].

We thus hypothesized that saaHDL, through SAA, could play a major role in the alteration of adipocyte metabolism, providing a molecular link between APR or low grade inflammatory disorders and associated lipid and glucose metabolism abnormalities.

## Methods

### Materials

Human recombinant SAA was purchased from PeproTech (Rocky Hill, NJ) and corresponds to human apoSAA1. Human HDL, SAA-enriched HDL and BAY 11–7082 were from Calbiochem. SAA content of SAA-enriched HDL was analyzed by SDS-PAGE and found to be in the range of 8–10% of total proteins. SAA and SAA-enriched HDL endotoxin content was assessed using the LAL assay from Lonza and found to be less that 0.1 ng per μg (0.1 EU/μg). [γ-^32^P]ATP was from Amersham Life Sciences. Primary antibodies for perilipin were from Progen Biotechnik. All other primary antibodies and MEK1/2 inhibitor PD95059 were from Cell Signaling. Secondary antibodies were obtained from Rockland. All other chemicals were from Sigma, including SB203580, SP600125 and H-89 inhibitors. For western-blots, molecular weights were calculated by interpolation from known standards (Bio-rad, München, Germany).

### Cell culture

Adipocyte differentiation of Multipotent Adipose-Derived Stem cells isolated from human adipose tissue (hMADS) was performed as described previously [24], [25] with the following modifications: 1) unless stated, cells were cultivated in 96-well plates, 2) at post-confluence, cells were induced into adipogenic differentiation through DMEM/Ham F12 media supplemented with 0.86 μM insulin, 0.2 nM T3, 10 µg/mL transferrin, 1 μM dexamethasone (DEX), 100 μM IBMX and 200 nM GW7845, a PPARγ agonist. Three days later, the medium was changed and supplemented with insulin, T3 and transferrin. Cells were used from day 7 to day 12 post-induction.

Subcutaneous abdominal adipose tissue was obtained from nondiabetic subjects who underwent plastic abdominal surgery at the Department of General Surgery, St Louis hospital, Paris, France. Samples were collected with the approval of the St Louis Ethics Committee and all subjects gave their written consent. Subjects on endocrine therapy or antihypertensive therapy and patients with malignant diseases were excluded. Mature adipocytes were isolated by the flotation method following 1h of collagenase (Roche) digestion and several washes. 3.10^6^ ells in DMEM 4.5 g/L glucose with L-Glutamine, sodium pyruvate, 1 g/L BSA were used within 24 h.

### Adipocyte assays

Cytotoxicity was measured with the Lactate Dehydrogenase Assay (Cayman). Lipolysis was evaluated by measuring glycerol release from adipocytes using a Glycerol Colorimetric kit (RANDOX®). Adipocytes were incubated in DMEM/F12 supplemented with 2 g/L low endotoxin BSA. Secreted adipokines and cytokines were measured using ELISA kits according to the manufacturers' instructions (human MCP-1 and IL-6 kits: Bender Medsystems; human IL-8, adiponectin and leptin).

### RNA preparation and Real time quantitative PCR (RTqPCR)

Total RNA was isolated using ABIPRISM 6100® (Applied Biosystems), except for isolated mature adipocytes for which Total RNA Isolation Kit (Macherey Nagel) was used. Levels of mRNA were assessed by RTqPCR as described previously [26]. Oligonucleotides are given in Table S1. Data were normalized using LRP10 RNA [27].

### NF-κB and MAP Kinases signaling studies:

Upon stimulation with SAA (10 µg/mL), hMADS adipocytes cultured in 6-well plates were rinsed briefly with ice-cold PBS. The cells were scraped in 300 µL lysis buffer (Cell Signaling). The lysates were passed through a 25-gauge needle and centrifuged at 20,000×*g* for 5 min at 4°C. Protein extracts were resolved on 4–12% SDS-PAGE gel and transferred onto nitrocellulose membranes. The fluorescent signals were detected using Odyssey software system (v.2.1, Li-Cor). Electrophoretic Mobility Shift Assays (EMSA) was performed as described previously [26].

### Statistical Analysis:

Results are shown as means ± S.D. Statistical significance was determined using the Student's *t*-test. Significance was set at *p*<0.05. (Statistical significance: * *p*<0.05; ** *p*<0.01; ***p<0.001)

## Results

### SAA and saaHDL stimulate lipolysis

As already shown in human and porcine adipocytes, we found that SAA and SAA-enriched HDL (saaHDL) were able to stimulate lipolysis by a mechanism involving the phosphorylation of HSL (results not shown).

### SAA and saaHDL induce the secretion of cytokines and chemokines but reduce adiponectin secretion

**Figure 1:**
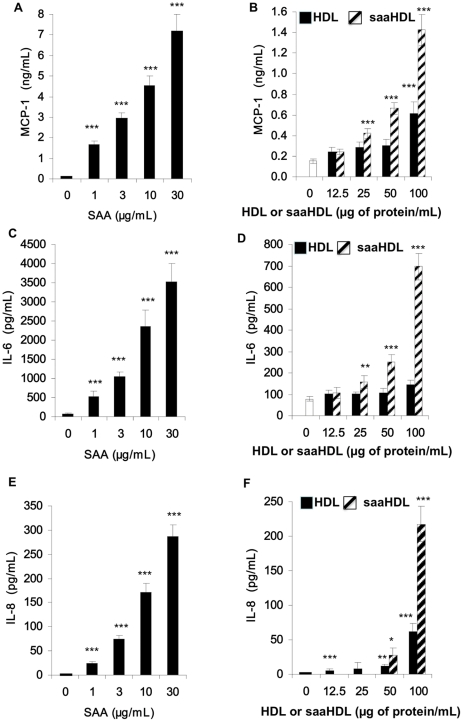
SAA and saaHDL induce pro-inflammatory adipokine secretion by hMADS adipocytes . hMADS adipocytes were cultured in presence or absence of human recombinant SAA (1, 3, 10 and 30 µg/mL), humans HDL and saaHDL (12.5, 25, 50 and 100 µg/mL) for 24 h. Upon treatment, the supernatants were recovered and secreted concentrations of MCP-1 (Panels A and B), IL-6 (Panels C and D) and IL-8 (Panels E and F) were measured by ELISA. Data are expressed as mean ± SD from n = 3–4 independent experiments. Statistical significance: * *p*<0.05; ** *p*<0.01, *** p<0.001 vs. control.

One important aspect of adipocyte function is the secretion of a number of anti- or proinflammatory molecules. We then examined the effects of both SAA and saaHDL on cytokine and chemokine secretion in hMADS adipocytes following a 24 h treatment. SAA dose-dependently induced MCP-1, IL-6 and IL-8 secretion (Figure 1, panels A, C, E) while having no effect on TNFα and SAA secretion (data not shown). Similar effects were observed for saaHDL although the magnitude of secretion was lower at the equivalent SAA dose of 10 µg/mL (Figure 1, panels B, D, F). We also observed a slight increase in MCP-1 and IL-8 secretion with increasing doses of HDL. This effect was not observed for IL-6.

**Figure 2:**
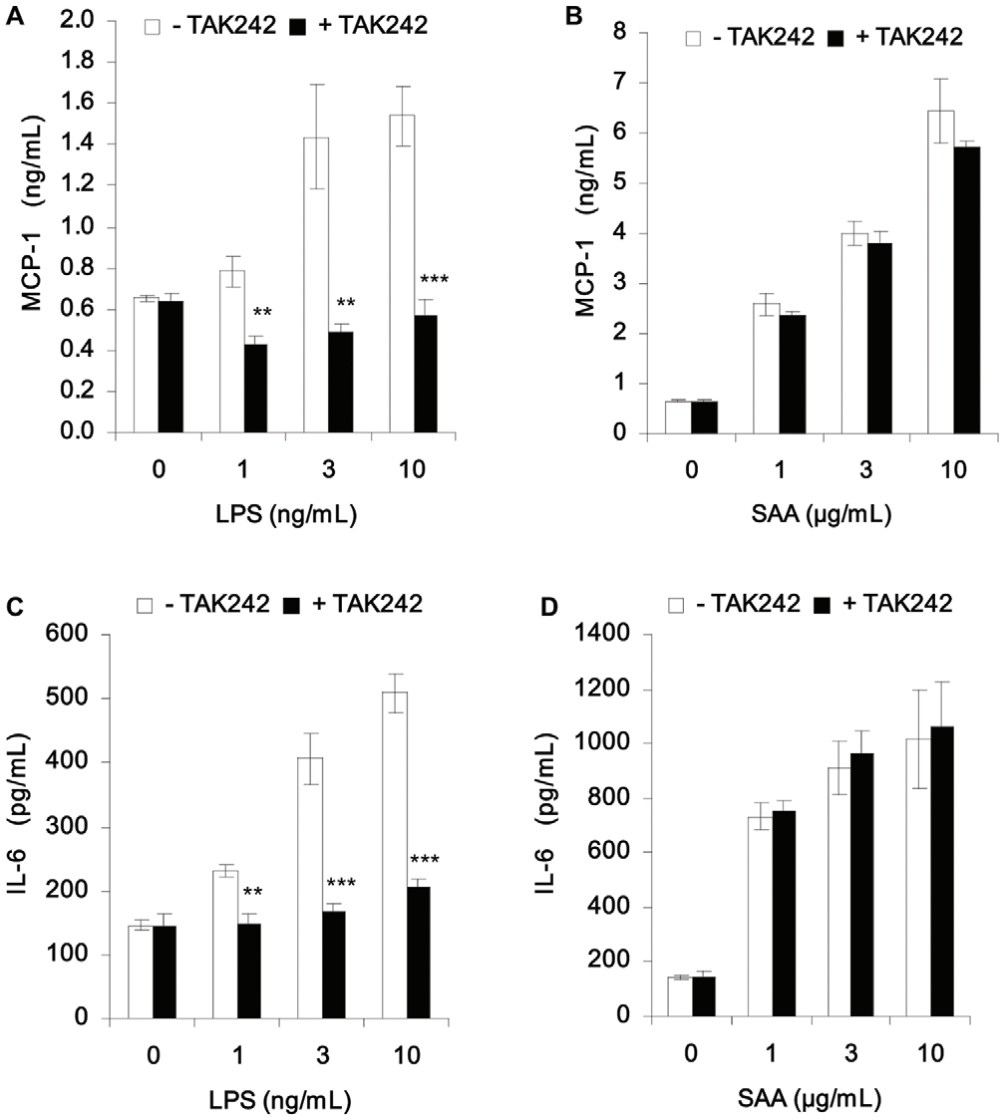
SAA pro-inflammatory effects in hMADS adipocytes are not due to endotoxin contamination. Fully differentiated hMADS adipocytes were preincubated with or without 2 µM TAK-242 inhibitor for 1 h. Upon preincubation, cells were treated in the presence or absence of recombinant human SAA (1, 3 and 10 µg/mL) or LPS (1, 3 and 10 ng/mL) for 24 h. At the end of the treatment period, the supernatants were recovered and secreted MCP-1 (Panels A and B) and IL-6 (Panels C and D) were measured by ELISA. Data are expressed as mean ± SD from 3 independent experiments.

In order to rule out the contribution of a potential SAA endotoxin contamination to the stimulation of cytokines and chemokines secreted by hMADS adipocytes, we have tested the effect of SAA in the presence of a TLR-4 signaling inhibitor, TAK-242 [28]–[29] and compare it to the effect of LPS (lipopolysaccharide). TAK-242 completely abolished LPS-induced MCP-1 and IL-6 secretion (Figure 2, panels A, C) whereas it had no effect on SAA-induced MCP-1 and IL-6 secretion (Figure 2, panels B,D). Therefore, these results confirm that the induction of pro-inflammatory cytokines and chemokines by SAA is not due to trace amounts of endotoxin in the recombinant human SAA.

**Figure 3:**
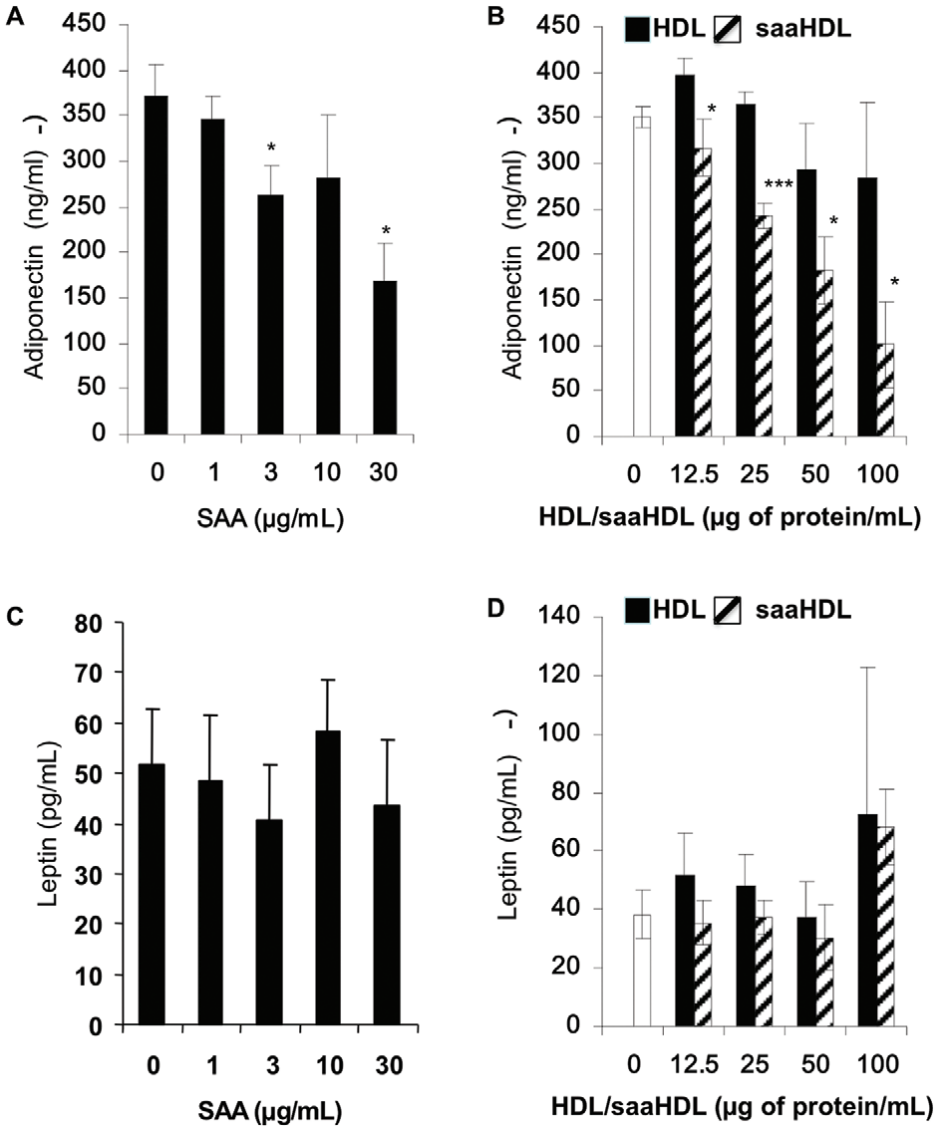
SAA and saaHDL decrease adiponectin secretion by hMADS adipocytes. hMADS adipocytes were cultured in the presence or absence of human recombinant SAA (1, 3, 10 and 30 µg/mL), humans HDL and saaHDL (12.5, 25, 50 and 100 µg/mL) for 3 days following which secreted adiponectin (Panels A and B) and leptin (Panels C and D) were measured by ELISA. Data are expressed as mean ± SD from 3 independent experiments. Statistical significance: * *p*<0.05; *** *p*<0.001 vs. control.

A 24 h SAA treatment did not modify adiponectin secretion (data not shown). However, upon 72 h treatment we observed a dose-dependent decrease in adiponectin secretion (up to 55% inhibition at 30 µg/mL) (Figure 3, panel A) whereas leptin secretion was not affected (Figure 3, panel C). Similar effects were observed using saaHDL compared to normal HDL with a 70% decrease in adiponectin secretion and no effect on leptin secretion (Figure 3, panels B and D).

### SAA modifies the expression of adipocyte gene

**Figure 4:**
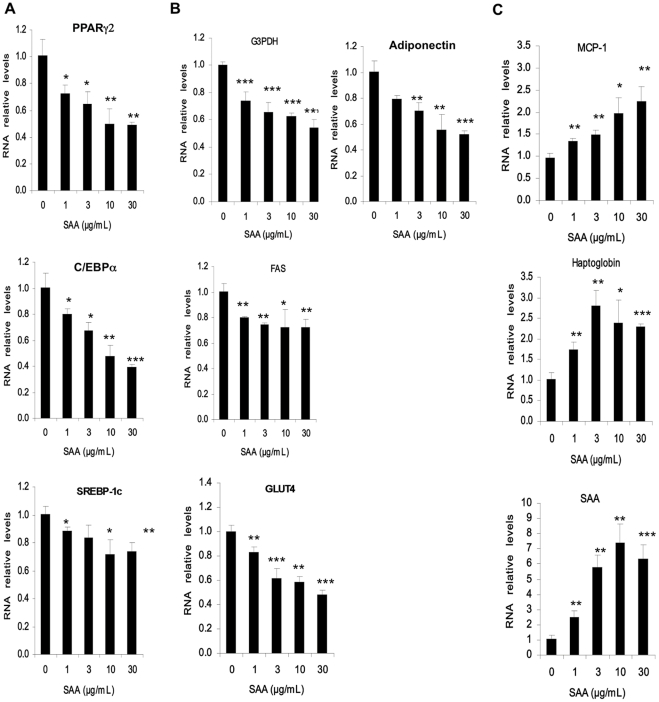
SAA decreases the expression of adipocyte markers. hMADS adipocytes were cultured in the presence or absence of human recombinant SAA (1, 3, 10 and 30 µg/mL) for 24 h. Upon treatment, total RNA was extracted and expression levels of various genes were analyzed by RTqPCR. Panel A: three important transcription factor (PPARγ2, C/EBPα and SREBP-1c). Panel B: lipogenic genes and adiponectin. Panel C: inflammation related proteins. The mRNA levels, normalized to LRP10 RNA expression, were determined relative to untreated control cells. Data are expressed as mean ± SD from n = 3–5 independent experiments. Statistical significance: * *p*<0.05; ** *p*<0.01; *** *p*<0.001 vs. control.

To determine whether SAA-mediated alterations of adipocyte metabolism were concomitant with changes in adipogenic gene expression, we examined the effect of SAA on the expression of two adipocyte transcription factors involved in adipocyte differentiation, Peroxisome Proliferator Activated Receptor-gamma 2 (PPARγ2, and CCAAT/Enhancer Binding Protein alpha (C/EBPα and one involved in lipid synthesis, Sterol Regulatory Element Binding Protein-1c (SREBP-1c). We also measured the expression of several of their target genes. Dose-dependent decreases in the expression of these three genes were apparent after a 24 h treatment of hMADS with SAA (Figure 4, Panel A). The mRNA levels of PPARγ2 and SREBP-1c target genes such as adiponectin, Glyceraldehyde 3-Phosphate Dehydrogenase (G3PDH) and Fatty Acid Synthase (FAS) were also repressed (Figure 4, Panel B). The mRNA levels of the glucose transporter GLUT4 were downregulated (50% inhibition at 30 µg/mL) (Figure 4, Panel B), potentially inducing a decreased insulin stimulated glucose transport in adipocytes. In contrast, insulin receptor expression was not affected (results not shown). We finally looked at the mRNA levels of Monocyte Chemoattractant Protein-1 (MCP-1), SAA, haptoglobin and Plasminogen Activator Inhibitor-1 (PAI-1). With the exception of PAI-1 (data not shown), mRNA levels of other adipokines (MCP-1, Haptoglobin and SAA) were dose-dependently increased (Figure 4, Panel C).

### SAA activates NF-κB and MAPK signaling pathways

**Figure 5:**
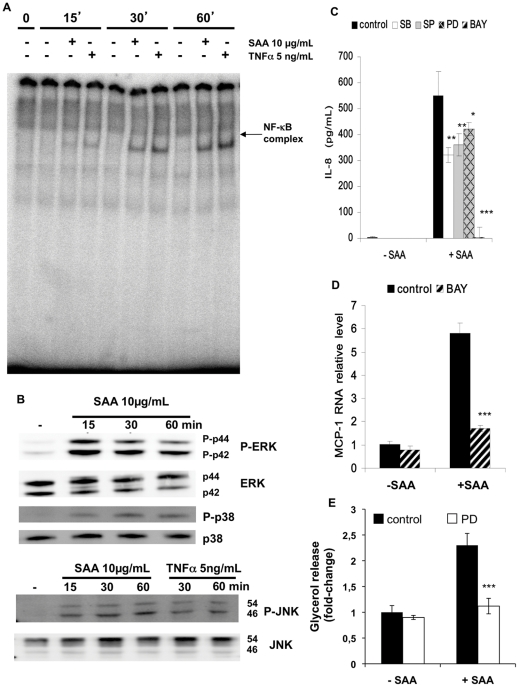
SAA **activates NF-κB and MAPK pathways and NF-κB inhibition suppresses inflammation.** hMDAS adipocytes were stimulated with 2 µg/mL recombinant human SAA or 5 ng/mL TNFα (positive control) for different periods of time. Nuclear proteins were extracted and EMSA performed as described in “Materials and Methods”. The NF-κB/DNA complex was detected by [γ-^32^P]-labeled NF-κB probe (Panel A). Total proteins were also extracted to determine total and phosphorylated forms of MAP kinases, p44/42, p38 and SAPK/JNK (Panel B). Following a 48 h insulin deprivation, hMADS adipocytes were incubated for 1 h with various inhibitors (10 µM) and subsequently incubated for 6 h and 24 h with SAA (10 µg/mL). SB203580, a p38 inhibitor; SP600125, a JNK inhibitor; PD98059, a MEK1/2 inhibitor; BAY11-7082, an inhibitor of IκB-alpha phosphorylation. At the end of the treatment period, the supernatants were recovered. Secreted IL-8 (Panel C) was measured by ELISA. MCP-1 gene expression was analyzed by RT-qPCR (Panel D). Glycerol was measured in the cell supernatant using a colorimetric assay (Panel E). * *p*<0.05; ** *p*<0.01; *** *p*<0.001 vs. control.

We then addressed the potential intracellular signalling pathways involved in the metabolic and gene alterations induced by SAA. Since SAA induces NF-κB and MAPK signaling pathways in immune cells, we examined these early signaling events by treating hMADS adipocytes with SAA (10 µg/mL) for up to 1 h. In order to determine whether SAA activates the NF-κB pathway, an electromobility shift assay was performed using nuclear extracts of hMADs adipocytes treated with SAA or with TNFα as a positive control. SAA stimulated the translocation of the NF-κB complex in the nuclear compartment as early as 15 min in a way very similar to TNFα (Figure 5, panel A). We next examined the phosphorylation status of MAPK (p44/42, JNK and p38). SAA induced p44/42, p38, and p46/54-JNK phosphorylation (Figure 5, panel B). A similar response for the phosphorylation of p46/54-JNK was observed using TNFα as shown previously in macrophages [30]. In conclusion, SAA is able to induce NF-κB and MAPK signaling pathways in human adipocytes.

### Inflammation induced by SAA is blocked by inhibiting the NF-κB pathway

To determine whether the induction of proinflammatory factors is a consequence of the activation of these signaling pathways, we used SB203580, a p38 inhibitor, PD98059, a MEK1/2 inhibitor, SP600125, a JNK inhibitor and BAY11-7082, an inhibitor of IκB-alpha phosphorylation. Only BAY11-7082 (10 µM), a JNK inhibitor was able to inhibit IL-8 secretion induced by SAA, IL-6 secretion (data not shown) and MCP-1expression (Figure 5, panel C and D). Cell viability assessed by LDH release was not affected by incubation with both SAA and the various inhibitors (results not shown).

### SAA effect on lipolysis is blocked by inhibiting the ERK pathway

While BAY11-7082 blunted SAA-induced MCP-1 gene expression (Figure 5, panel D), it failed both to prevent SAA stimulated lipolysis and to restore PPARγ2, C/EBPα and SREBP-1 c gene expression (data not shown) suggesting that the metabolic effects induced by SAA are direct and not an indirect consequence of its inflammatory effects. The various inhibibitors were unable to antagonize the lipolytic effects of SAA except for the MEK-1 inhibitor which completely blunted SAA stimulated lipolysis (Figure 5, panel E and results not shown) indicating that as previously described [23], SAA stimulates lipolysis through the ERK pathway.

### SAA induces inflammation and decreases adipocyte markers in freshly isolated mature adipocytes

**Figure 6:**
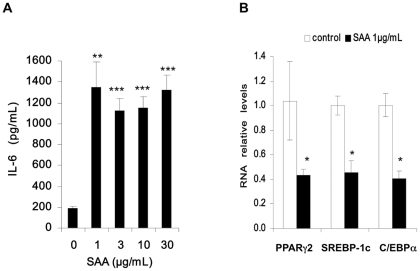
SAA exerts pro-inflammatory effects in adipocytes isolated from human subcutaneous adipose tissue. Isolated human adipocytes were prepared as described in “Methods” and were cultured for 24 h in presence or absence of human recombinant SAA (1, 3, 10 and 30 µg/mL). Secreted IL-6 (Panel A) was measured by ELISA. Gene expression levels of PPARγ2, C/EBPα and SREBP-1c were analyzed by RT-qPCR (Panel B). The mRNA levels normalized to LRP10 RNA expression were determined relative to untreated control cells. Statistical significance: * *p*<0.05; ** *p*<0.01; *** *p*<0.001 vs. control for three preparations.

In order to confirm the effects of SAA in human adipocytes, we have analysed the effects of SAA on freshly isolated mature human adipocytes from subcutaneous adipose tissue following a 24 h treatment. A low dose (1 µg/mL) of SAA resulted in a maximal induction of IL-6 secretion (Figure 6, panel A) as well as IL-8 and MCP-1(data not shown) and suppression of PPARγ2, C/EBPα and SREBP-1c (Figure 6, panel B), showing that the action of SAA is not restricted to the adipocyte cell line.

## Discussion

In this study, we demonstrate that beside its effects on cholesterol metabolism and transport [10], SAA has direct effects on adipocytes, which can contribute to the overall metabolic and cytokine changes observed during APR. Although SAA concentration during APR can reach concentrations as high as 1 mg/mL, we show here that at much lower concentrations (1 to 30 µg/mL), similar to those observed in obese patients [17], SAA has already clear-cut effects on adipocytes and could also contribute to the metabolic and endocrine characteristics of obesity.

During APR, there is a decrease in the capacity of adipose tissue to store FFAs. Our data suggests that the decrease in lipid content is a consequence of several effects of SAA on adipocytes such as 1) decrease in gene expression of transcription factors important for adipocyte storage such as PPARγ2, C/EBPαand SREBP-1c; 2) decrease in gene expression of the main enzymes involved in lipogenesis like G3PDH and FAS; 3) increase in lipolysis through activation of the ERK pathway and HSL as previously described also in other models [22], [23]. Since SAA strongly reduces the expression of the glucose transporter GLUT4 in adipocytes, a decreased fatty acid storage capacity could be also the consequence of a reduced glucose uptake since glucose is necesssary for the synthesis of α-glycerophosphate, which forms the backbone of triglycerides. It remains to show however that insulin-induced glucose transport is indeed affected by SAA in adipocytes. A stimulated lipolysis and a decreased glucose transport in the presence of SAA by counteracting insulin action could ultimately participate to the impairment of adipose tissue insulin sensitivity [31] observed during APR.

Given that SAA is a pro-inflammatory cytokine on immune cells, we studied the adipokines secretion profile of human adipocytes [12]. We demonstrate that both saaHDL and SAA induce the expression and secretion of pro-inflammatory proteins such as MCP-1, IL-6 and IL-8, while adiponectin secretion is repressed. This secretion profile is the hallmark of hypertrophic adipocyte [32]. Several studies in obese humans have shown that adipose tissue expression of SAA appears to be restricted to adipocytes and in particular to hypertrophic adipocytes [17], [21]. Plasma SAA levels are also increased in human obesity in a range compatible with the metabolic effects observed in this study [17], [33], [34]. Therefore, besides the induction of changes in adipocyte metabolism by SAA during APR, pathophysiological exposure of adipocytes to SAA could directly contribute to the establishment of the inflammatory and insulin resistant phenotype in adipose tissue in obese patients [34].

In humans, SAA1 is mainly produced by the liver during the APR, while in obesity adipocytes contribute to the plasma SAA levels [35]–[37]. In murine models, SAA3 is upregulated by diet-induced obesity in adipocytes [38]–[40]. SAA3 has been shown recently to form a complex with hyaluronan suggesting that it is trapped in the extracellular matrix [41]. These complexes facilitate recruitment, adhesion and retention of monocyte-macrophages within adipose tissue, a characteristic of obese adipose tissue.

We previously reported that SAA is a player in the dialogue between hypertrophied adipocytes and macrophages through its regulation of adipocyte cholesterol efflux [42]. Taken together, our new data along with the published observation provide further evidence for a central role of SAA in adipocyte-macrophage cross-talks in obesity: 1) SAA increases IL-6 and IL-8 production by adipocytes as well as the chemokine MCP-1, which recruits circulating monocytes into the adipose tissue; 2) SAA increases IL-6, IL-8 and TNFα production by immune cells; 3) SAA displays direct chemoattractant activity in the presence or absence of hyaluronan complexes [15], [33], [41]–[44].

This is the first study to address the early SAA intracellular signaling pathways in human adipocytes which demonstrates that SAA activates NF-κB and MAPK signaling pathways (p44/42, p38 and p46-JNK) in these cells. Furthermore, BAY-117082, a NF-κB pathway inhibitor, completely blunted SAA-induced inflammation while PD98059, an ERK pathway inhibitor blunted SAA-induced lipolysis. A majority of studies has shown that SAA signals through a seven transmembrane G-coupled receptor FPRL1 leading to NF-κB and MAPK pathways activation whilst other reports suggest that SAA could also bind and signal through Scavenger Receptor class B type I (SR-BI/CLA-1) at least for MAPK activation [15], [44]–[46]. In Rheumatoid Arthritis patients, SAA has been shown to bind and activate the Receptor for Advanced Glycation End-products (RAGE) [47]. Some studies have suggested that TLR4 may mediate SAA signaling [48]. However given the fact that TAK-242, a TLR-4 signaling inhibitor did not block SAA mediated inflammation in adipocytes, it is unlikely that TLR4 is involved in SAA signaling in adipocytes. A fourth SAA receptor has also been suggested: Tanis/SelS whose expression has been linked to glucose and triglyceride levels in an animal model of type 2 diabetes and metabolic syndrome *Psammomys obesus* [49], could be a candidate for the metabolic effects of SAA in human adipocytes. However, the receptor(s) mediating the effect of SAA in adipocytes still remain to be identified.

We have extended these results to mature human adipocytes freshly isolated from subcutaneous adipose tissue. In mature human adipocytes, a low dose of SAA (1 µg/mL) was able to confer a maximal induction of inflammation as well as a maximal repression of the transcription factors (PPARγ2, C/EBPα and SREBP-1c) suggesting higher SAA receptor expression.

In summary, our data suggest that SAA could be at least partly responsible for the metabolic changes of the adipose tissue during the APR. At the molecular level, SAA represses the gene expression of PPARγ2, C/EBPα and SREBP-1c, three important transcription factors. In addition, SAA activates the NF-κB pathway leading to the induction of inflammation. These effects could translate into a phenotype characterized by a coordinated decrease in the storage of FFAs and an increase in FFAs mobilization and at least partly explain the reduced insulin efficiency concomitant with APR. Our findings also reveal that in conditions of low grade inflammation such as obesity, SAA could participate to the metabolic phenotype characterized by adipose tissue inflammation, insulin resistance and fatty acid overflow from adipocytes.

## Supporting Information

Table S1 Forward and reverse oligonucleotides used for Real Time quantitative PCR.
(TIF)
