# Supplementary figures and images for: The Acute Phase Protein Serum Amyloid A Induces Lipolysis and Inflammation in Human Adipocytes through Distinct Pathways
Source: PLoS One. 2012 Apr 19;7(4):e34031. doi: 10.1371/journal.pone.0034031 (PMC3331860; doi:10.1371/journal.pone.0034031)

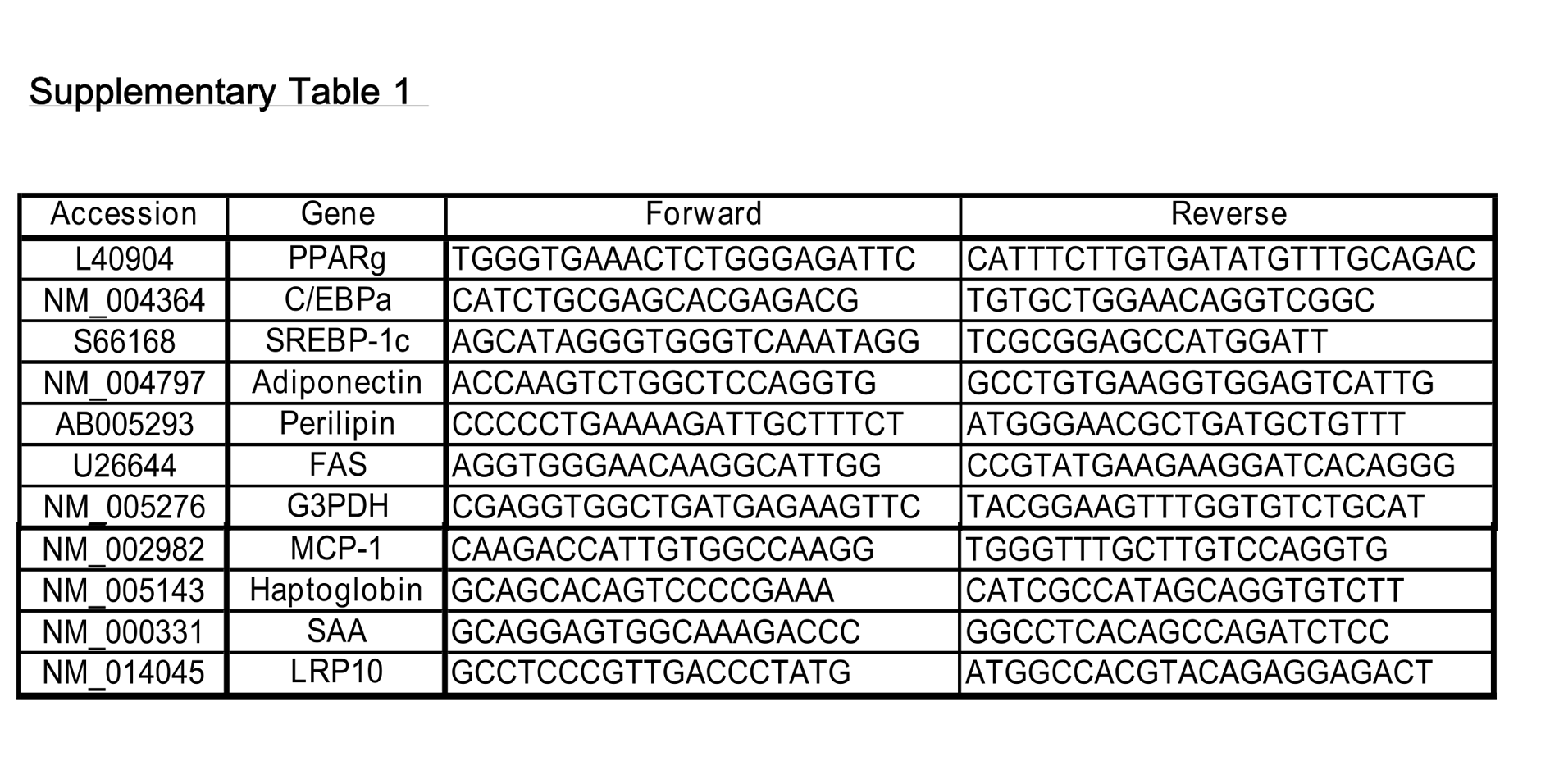

Supplement: Table S1 — Forward and reverse oligonucleotides used for Real Time quantitative PCR. (TIF) [file pone.0034031.s001.tif]
